# Supplementary figures and images for: AgRP neuron activity predicts and tracks the glycemic response to oral glucose
Source: Mol Metab. 2026 Apr 17;108:102372. doi: 10.1016/j.molmet.2026.102372 (PMC13141783; doi:10.1016/j.molmet.2026.102372)

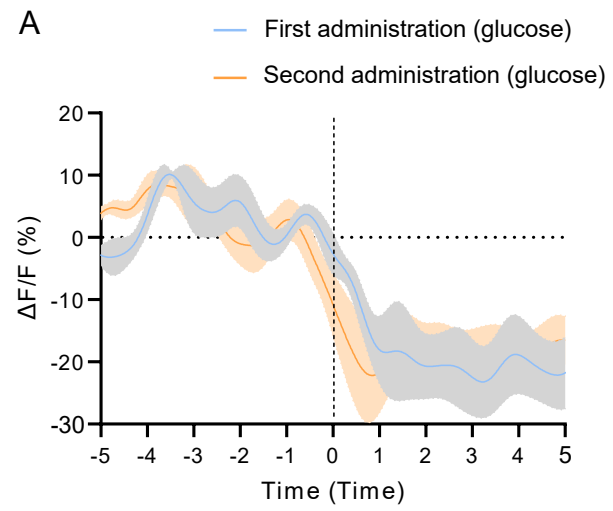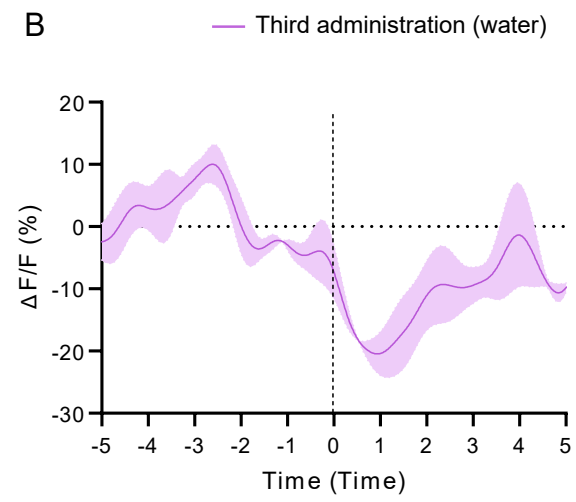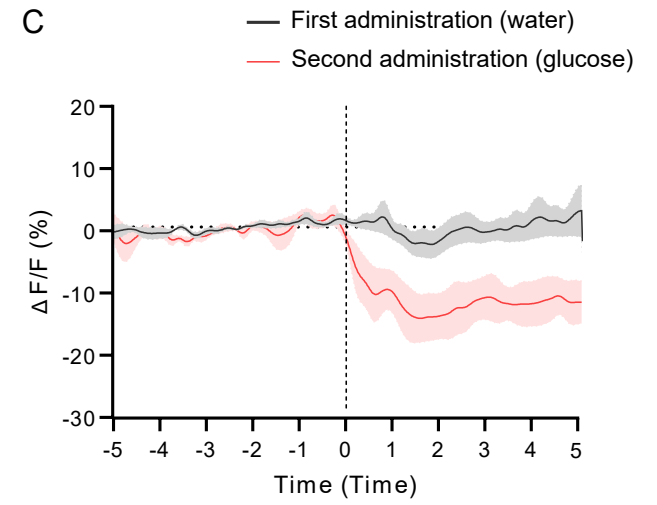

Supplement: Figure S1 — AgRP neurons exhibit anticipatory responses to glucose administration via OG. (A) Mean AgRP neuron responses to the first and second OG glucose (2 g/kg) (n = 3). (B) Mean AgRP neuron activity when water was administered after prior glucose exposures (n = 3). (C) AgRP neuron activity when water was given as the first OG, followed by 2.0 g/kg glucose on the subsequent gavage (n = 3). [file mmc1.pdf]

A

AgRP activity

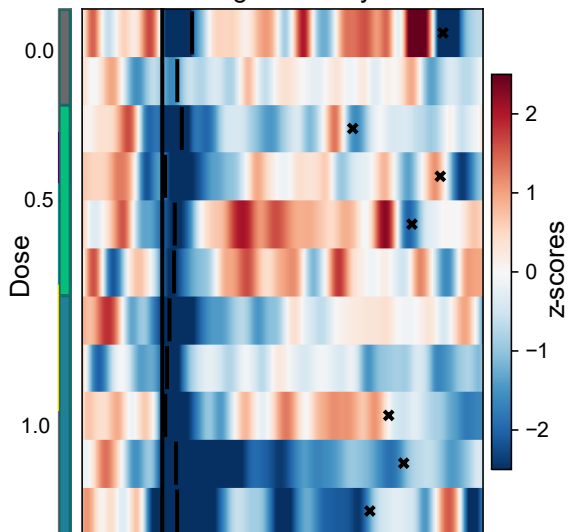

B

Early model prediction

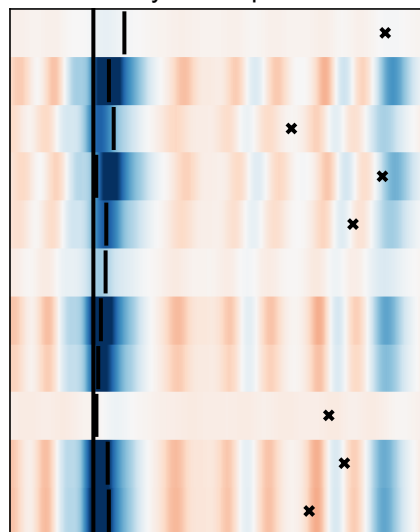

C

Residual

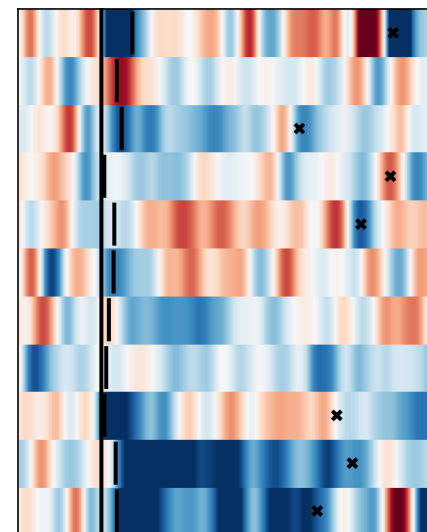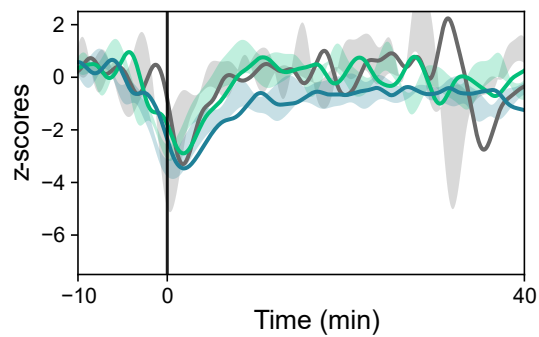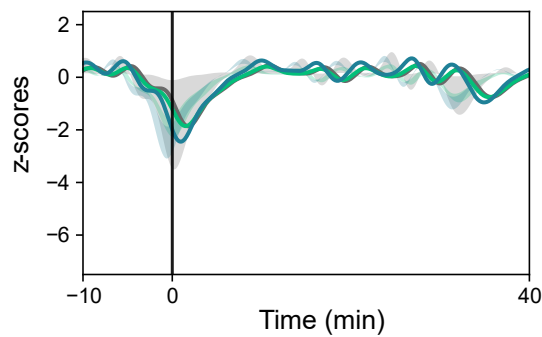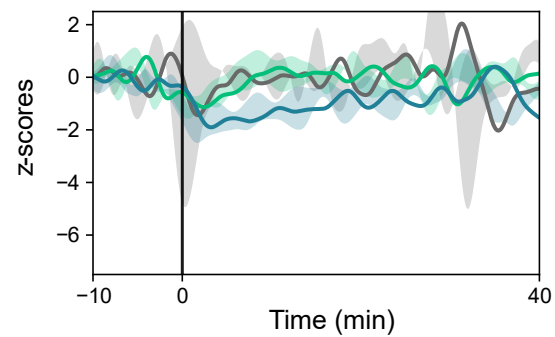

Supplement: Figure S2 — Dissociation between phase 1 and phase 2 responses using an early-response model in low doses of glucose. (A-C), Heat-maps (top) and mean AgRP neuron activity (bottom) for the original (A), fitted (B), and residual (C) traces in mice administered with water (0.0), 0.5, or 1.0 g/kg glucose. [file mmc2.pdf]

A

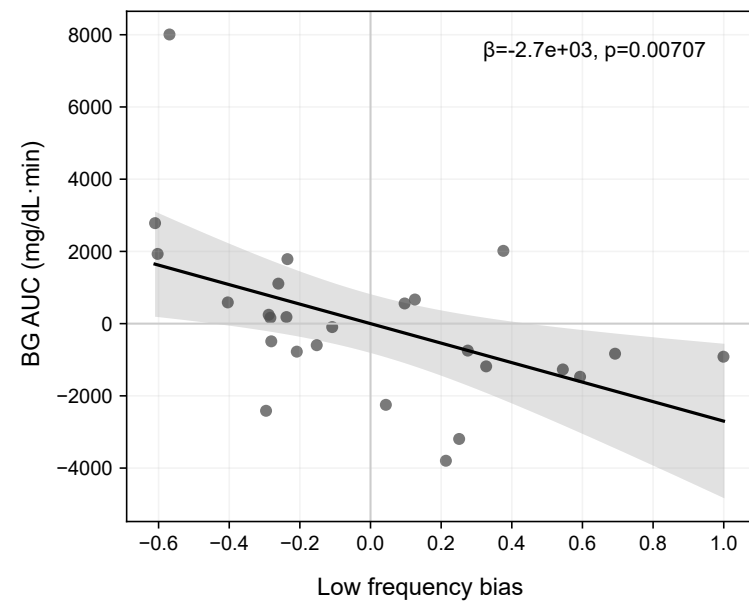

Supplement: Figure S3 — Relationship between baseline low-frequency power and subsequent BG dynamics. (A) Relationship between individual session aperiodic slope in AgRP neuron activity (higher slope à greater low-frequency bias in baseline period) and incremental AUC of BG level. Slope from linear mixed effects model with corrections for OG dose and subject. [file mmc3.pdf]

**A**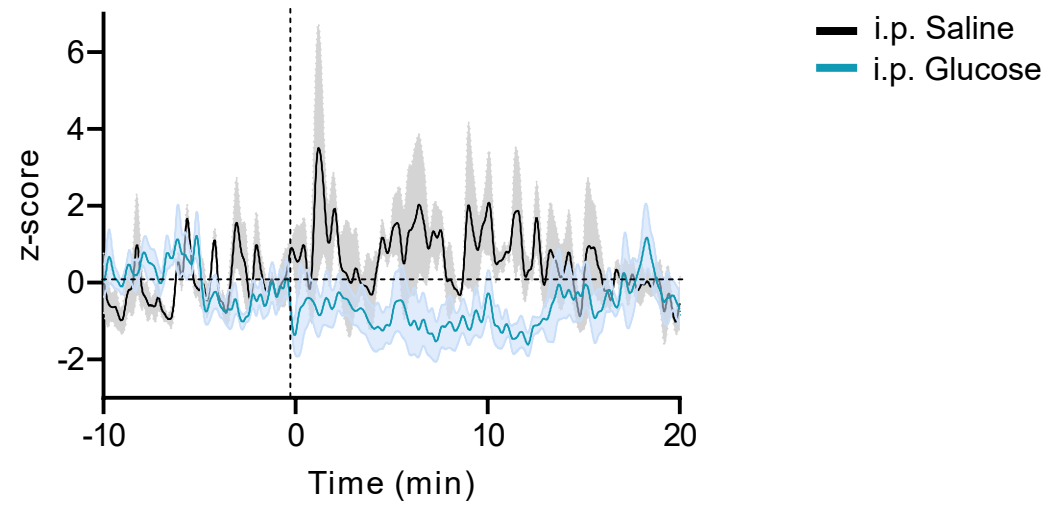**B**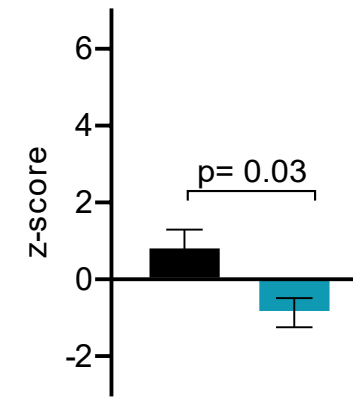

Supplement: Figure S4 — AgRP neuron inhibition following i.p. injection of glucose (2 g/kg) or saline. (A) GCaMP6s responses to i.p. glucose (2 g/kg) or saline in 5-hour fasted mice. (B) Quantification of z-score from (A) over the 0–15 min period following injection. Mann–Whitney test, p = 0.03, glucose: n = 5; saline: n = 3. [file mmc4.pdf]
